# Alterations in corneal epithelial dendritic cell in Sjogren’s syndrome dry eye and clinical correlations

**DOI:** 10.1038/s41598-022-15537-4

**Published:** 2022-07-01

**Authors:** Ran Hao, Yi Ding, Xuemin Li

**Affiliations:** 1 Department of Ophthalmology, Peking University Third Hospital, No. 49, North Garden Street, Beijing, China; 2 Beijing Key Laboratory of Restoration of Damaged Ocular Nerve, Peking University Third Hospital, No. 49 North Garden Road, Haidian District, Beijing, China; 3 Capital Medical University, No.10, Xi Toutiao, Youanmen Wai Street, Beijing, China

**Keywords:** Eye diseases, Autoimmunity, Inflammation

## Abstract

We aimed to investigate the density and morphology of corneal dendritic cells (DCs) in dry eye (DE) patients with or without Sjogren’s syndrome (SS). This study included 28 patients with Sjogren’s syndrome dry eye (SSDE), 33 patients with non-Sjogren’s syndrome dry eye (NSSDE), and 30 age and sex matched healthy volunteers. In vivo confocal microscopy (IVCM) was used to investigate density and morphology (size, dendrites, and field) of DC. Compared with NSSDE and healthy group, SSDE showed significantly higher DC density, larger DC size, more DC dendrites with larger DC field (all *P* < 0.001). Comparison between NSSDE and healthy group demonstrated that DC density, dendrites and field were significantly higher in NSSDE. However, there was no significant difference in DC size (*P* = 0.076). DC density and morphological parameters showed significant associations with the systemic severity (salivary gland biopsy and serum antibodies) and ocular surface damage. The corneal epithelium DC density and morphological alterations were obvious in SSDE, which reflected higher level of immune activation and inflammatory response in SS. Marked correlations were found between DC density/morphology and systemic/ocular severity. Dynamic assessment of corneal DC may facilitate to clarify pathogenesis, stratify patient, and tailor treatment in SS patients.

## Introduction

Dry eye disease (DED) is one of the most frequent ophthalmic multifactorial disorders, which is assigned to aqueous tear deficient dry eye (DE) and evaporative DE by mechanism^1^. Additionally, the most common form, aqueous tear deficient DE, can be subdivided into Sjogren’s syndrome dry eye (SSDE), associated with a progressive autoimmune disease characterized by chronic lymphocyte infiltration of the salivary and lacrimal glands, and non-Sjogren’s syndrome dry eye (NSSDE), tear deficiency by other causes^1,2^. The SSDE patients typically have been suffering terrible xerophthalmia and ocular surface injury for a long time, with multiple organ dysfunction and even under the threat of blindness and death (because of multiple organ failure and non-Hodgkin’s lymphoma)^1,3,4^. Therefore, early diagnosis and appropriate treatment play important roles in SSDE management.

DED is characterized by tear film instability, visual disturbance, inflammation and damage of the ocular surface^1,4,5^. Recent researches have shown that immune and inflammation play key roles in the pathogenesis of DED^4,6^, especially in SSDE and lead to severe xerophthalmia and ocular surface damage^6,7^. There is an increasing evidence that antigen-presenting cells (APCs), especially corneal dendritic cells (DCs) which are equipped to induce T-cell activation and inflammatory cascade, are crucial for the DED pathogenesis^8–11^. With the help of in vivo confocal microscopy (IVCM), the density and morphology of DCs in DED have been identified and provide a better insight to the pathogenesis of clinical manifestations^6,7^.

In patients with Sjogren’s syndrome (SS), DCs have been found to infiltrate into the lacrimal and salivary glands in an ex vivo study^12^. Some studies also demonstrated increased DCs in the central corneal epithelium of SSDE^6,7^. Besides quantity, DCs morphology changes (such as size, dendrites number and length) are other biomarkers in response to inflammation and immunity^13–15^. Nevertheless, these studies have limitations such as small sample sizes and non-detailed DC parameters. Moreover, the correlations between the DC parameters and systematic/ocular characteristics may aid in the pathogenesis elucidation and severity assessment of SS. But there was no study has clarified the associations between the DC and serum antibodies/salivary gland biopsy in SS. We aimed to evaluate the DC density and morphology (including size, dendrite and field) of the central corneal epithelium in normal subjects and DE patients with or without SS. Such a determination was made to clarify whether there was an association among these parameters and the pathogenesis and severity of SS.

## Materials and methods

### Participants and clinical study

Sixty-one consecutive patients with aqueous tear deficient DE, including 28 with SSDE and 33 with NSSDE, and 30 healthy volunteers were enrolled. Only the right eye was evaluated. The study was performed according to the principles of the Declaration of Helsinki and was approved by the Human Research and Ethics Committee, Peking University Third Hospital (No. M2019236). Written informed consent was obtained from each study participant.

DED was defined according to the following international diagnostic criteria by the Dry Eye WorkShop II (DEWS II) of the Tear Film and Ocular Surface Society (TFOS): ocular surface disease index (OSDI) ≥ 13 and tear break-up time (TBUT) < 10 s or ocular surface staining (> 5 corneal spots and > 9 conjunctival spots)^1,16^. The diagnosis of SS was made according to the American College of Rheumatology-European League Against Rheumatism (ACR-EULAR) criteria^3^. The DED patients without SS were categorized to NSSDE group^1,16^. The healthy controls were asymptomatic participants without any ocular injury and medication, with normal tear functions and negative ocular surface staining (OSS)^16^. The exclusion criteria included the systemic/ocular disease except for DE and SS, especially ocular inflammation (such as infection and allergy), rheumatoid arthritis, systemic lupus erythematosus and other diseases which might affect DC density and morphology, history of contact lenses wear and ocular surgery, diabetes mellitus, and pregnancy. Since the anti-inflammatory medications are likely affecting the ocular surface, the patients enrolled in the study have not been treated with these medications.

A careful case history and the OSDI were recorded for each participant. Schirmer-I test (ST), tear meniscus height (TMH), TBUT and OSS score were performed to evaluate aqueous production, tear film stability and ocular damage using standard protocols^1,16^. The TMH was recorded with a Keratograph 5 M (OCULUS, Wetzlar, Germany) and repeated three times, then the average value was analyzed. The OSS score (0–12) for each eye was the summation of the fluorescein score for the cornea and the lissamine green score for the nasal and temporal bulbar conjunctiva^16^. Serum levels of anti-SSA52, anti-SSA, anti-SSB antibodies and salivary gland biopsy were assessed in DE patients according to the diagnostic criteria^3^. All salivary gland biopsies were performed by an experienced dentist and evaluated by the same experienced pathologist. Salivary gland biopsies were collected as follows^3,17^: (1) evert the lower lip to visualize the minor salivary glands; (2) inject 0.5–1.0 mL 1:100,000 1% lidocaine:epinephrine solution into the submucosa; (3) make a 1.0–1.5 cm linear incision, parallel to the vermilion border; (4) Blunt dissect on a plane perpendicular to the mucosal incision and parallel to the direction of the sensory nerve fibers; (5) recover 4–7 glands from each patient and store them in 10% formaldehyde; (6) close the incision with simple interrupted 4–0 chromic sutures. The salivary gland biopsies were stained with hematoxylin–eosin (HE) and analyzed under microscope. The result was considered positive if the focus score (defined as the number of mononuclear infiltrates containing ≥ 50 lymphocytes/4 mm^2^ of glandular tissue) was ≥ 1^3,17^. All examinations were carried out in the same examination room under the same conditions. The eye and systemic measurements were performed by the same masked observers.

### In vivo confocal microscopy

All participants had undergone imaging with a digital corneal confocal laser-scanning microscope of the central cornea using Heidelberg Retina Tomograph 3 with the sequence mode (HRT II RCM Heidelberg Engineering Inc, Heidelberg, Germany, Rostock Cornea Module). After topical anesthesia with 0.4% oxybuprocaine hydrochloride (Oftan Obucain, Santen Oy, Tampere, Finland). A drop of carbomer (Viscotears, CIBA Vision Europe Ltd., Southampton, UK) was served as a lubricant gel both in ocular surface and the disposable sterile polymethylmethacrylate cap (Tomo-Cap; Heidelberg Engineering GmbH, Dossenheim, Germany). Then advanced the cap manually until the gel contacted the central corneal surface as previously dsecribed^14,18^. The corneal sub-basal nerves shift centripetally and form a corneal vortex just inferior to the nose of the corneal apex^19,20^. The corneal vortex is a land mark of central cornea and easy to identify with IVCM^20^. At least 100 images of corneal sub-basal layer (approximately at a depth of 50–70 μm) were obtained in each eye by the same masked professional technician. According to previous literature, three non-overlapping images with optimal quality in the central cornea were chosen for corneal sub-basal nerve^21–23^ or DCs analysis^6,7,14,24^. Therefore, three images with the best focused and contrast, without motion or folds were chosen for analysis. DC density and morphology were determined for each image, the morphology parameters including DC size (represented the DC activation and scored based on the cell body area with processes number), number of dendrites per DC and DC field (area bounded within cell span and the length of dendritic processes).

A semiautomatic Java-based image processing software (ImageJ, National Institutes of Health, Bethesda, MD) dedicated to the tracing and quantification of elongated image structures, such as dendrites and neurons, were used to evaluate the density and morphologic parameters of DC in IVCM images. The Cell Count tool was used to count DC density and the average for three images was recorded. The 10 most representative DCs (the largest cell body with more dendrites) in three images were selected for morphologic analysis according to Kheirkhah et al. study^6^. The DC size was further scored based on a 3-point grading scale (1–3): 1 (globular cell: hyperreflective cell body without dendritic process), 2 (small cell body with two or fewer processes), 3 (large cell body with more than two processes)^25–27^. The number of dendrites per cell was calculated manually. The DC field was measured using a polygon joining the dendrite tips around each cell according to Kheirkhah et al. study^6^. Two masked observers assessed all parameters and the averaged values were calculated for further analysis. A third observer is needed if more than 10% difference between the two observers and the average of three values was recorded for analysis.

### Statistical analysis

Statistical analysis was performed using SPSS version 26.0 (IBM Corp., Armonk, New York, USA). The normality of the data distribution was verified by the Kolmogorov–Smirnov test. Continuous variables were described as the mean ± standard deviation (SD) or median with interquartile range. Categorical variables were expressed as frequencies and percentages. Continuous variables were compared with ANOVA or Kruskal–Wallis tests among three groups. Continuous data between two groups were compared using Least Significant Difference (LSD) tests or Mann–Whitney nonparametric U-tests. Categorical variables were compared with the Chi Square test. Spearman’s rank order correlation was performed to identify the potential factors related to the density and morphologic parameters of DC from the variables of age, sex, systemic characteristics, as well as dry eye symptoms and signs. Logistic regression analysis and multiple linear regression analysis were used to evaluate the validity of DC density and morphology (size, dendrites and field) compared with salivary gland biopsy/serum antibodies and ocular parameters (OSDI, ST, TMH, TBUT and OSS) in SSDE patients, respectively. Since the OSS did not show a continuous distribution, normality transition (a logarithmic transformation) was performed before regression analysis. A *P* value < 0.05 was considered significant for all comparisons. The sample size was calculated by Stata/SE 15.0 (Stata Corp., USA). According to previous literature^7^, the DC density in the central corneal epithelium of SSDE, NSSDE and healthy controls were 127.9 ± 23.7 cells/mm^2^, 89.8 ± 10.8 cells/mm^2^, and 34.9 ± 5.7 cells/mm^2^, respectively. The sample size of each group was determined to be 12 eyes (α = 0.05 and β = 0.10). However, small sample size may be difficult to further clarify the associations among these parameters and pathogenesis and severity of SS. Therefore, we enrolled 28 eyes in SSDE group, 30 eyes in NSSDE group and 30 eyes in healthy group.

## Results

### Clinical data

This study included 28 eyes of 28 patients with SSDE (26 females and 2 males), 33 eyes of 33 patients with NSSDE (23 females and 10 males) and 30 eyes of 30 healthy controls (21 females and 9 males). The mean age was 56.61 ± 15.01 years (range, 30–80 years) in the SSDE group, 50.41 ± 13.53 years (range, 27–75 years) in the NSSDE group, and 57.73 ± 8.42 years (range, 32–83 years) in the control group. The demographic data and systematic/ocular characteristics of the three groups have been presented in Table 1. There was no significant difference in age and sex among the three groups (*P* = 0.052 and *P* = 0.088, respectively). Compared with the NSSDE patients and the healthy controls, the participants in the SSDE group showed higher OSDI (67.88 ± 12.25) and OSS [4 (2, 9)] scores, and lower ST (1.43 ± 0.92), TMH (0.09 ± 0.02) and TBUT (1.94 ± 0.72) (all *P* < 0.001). The NSSDE patients also showed more serious OSDI score (35.12 ± 11.23 vs. 8.61 ± 1.71) and signs (ST, TMH, TBUT and OSS score) than the healthy controls (all *P* < 0.001).

**Table 1:** The demographics and clinical data of the three groups.

Item	SSDE (n = 28)	NSSDE (n = 33)	Control (n = 30)	*P*
**Sex**				
Male, n (%)	2 (7.14%)	10 (30.30%)	9 (30%)	0.052
Female, n (%)	26 (92.86%)	23 (69.70%)	21 (70%)	
Age (years)^#^	56.61 ± 15.01	50.41 ± 13.53	57.73 ± 8.42	0.088
OSDI^#^	67.88 ± 12.25	35.12 ± 11.23	8.61 ± 1.71	< 0.001*^†^
ST (mm)^#^	1.43 ± 0.92	3.44 ± 1.59	16.97 ± 2.68	< 0.001*^†^
TMH (mm)^#^	0.09 ± 0.02	0.16 ± 0.05	0.21 ± 0.07	< 0.001*^†^
TBUT(s)^#^	1.94 ± 0.72	4.08 ± 2.20	6.56 ± 3.13	< 0.001*^†^
OSS^$^	4 (2, 9)	0 (0, 3)	0 (0, 0)	< 0.001*^†^
Salivary gland biopsy, n (%)	26 (92.85%)	0 (0%)	/	< 0.001^†^
Anti-SSA52, n (%)	17 (60.71%)	0 (0%)	/	< 0.001^†^
Anti-SSA, n (%)	24 (85.71%)	0 (0%)	/	< 0.001^†^
Anti-SSB, n (%)	13 (46.43%)	0 (0%)	/	< 0.001^†^

*SSDE* Sjogren’s syndrome dry eye, *NSSDE* non-Sjogren’s syndrome dry eye, *OSDI* ocular surface disease index, *ST* Schirmer test, *TMH* tear meniscus height, *TBUT* tear film breakup time, *OSS* ocular staining score, *anti-SSA* anti-Sjogren's syndrome A, *anti-SSB* anti-Sjogren's syndrome B.

^#^Mean ± standard deviation; ^$^Median (25% quantile, 75% quantile).

*P < 0.05 among the three groups; ^†^*P* < 0.05 between SSDE and NSSDE.

### DCs density and morphology among SSDE, NSSDE and Controls

**Figure 1:**
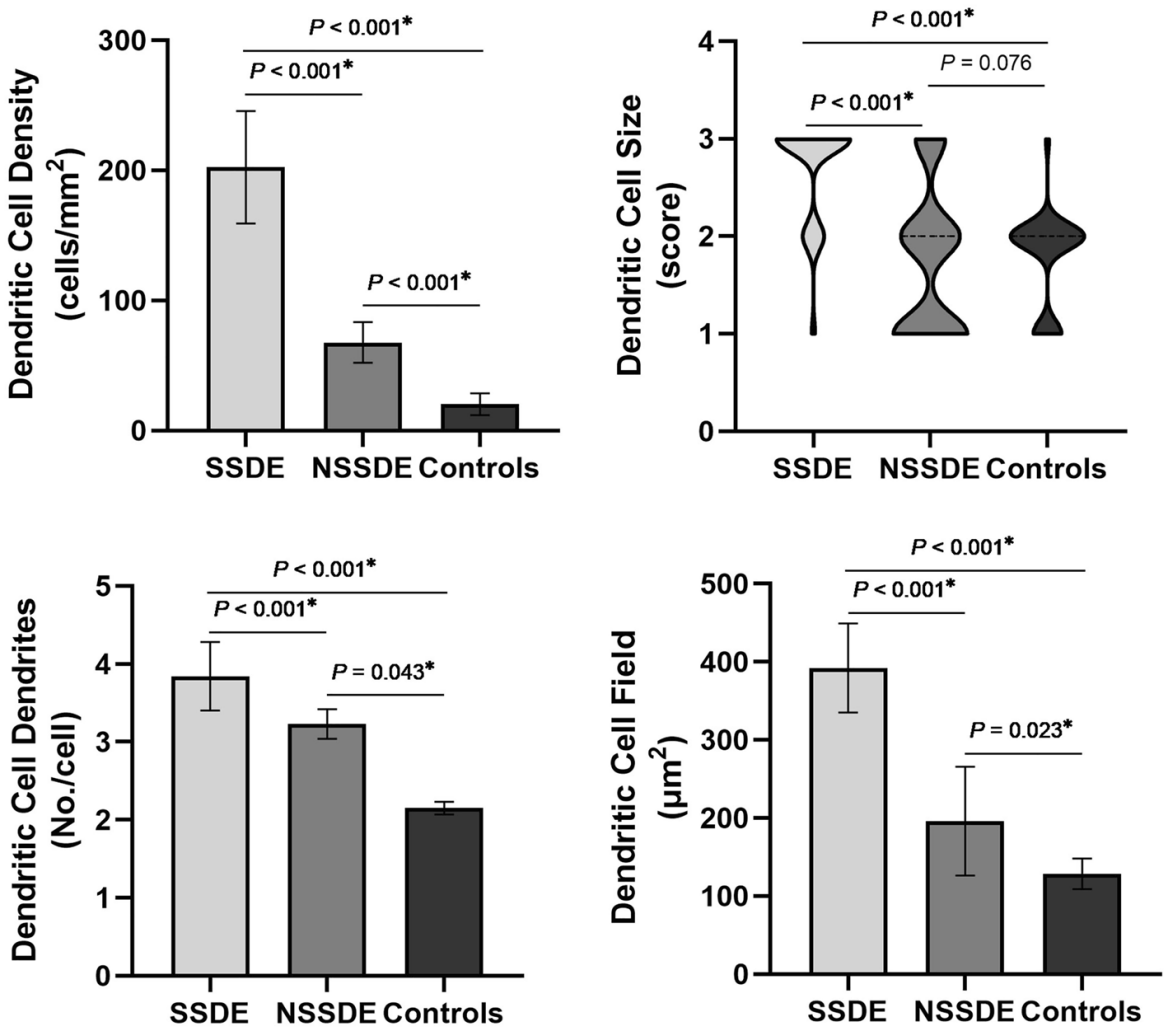
Dendritic cell density and morphologic parameters in SSDE, NSSDE and healthy controls. Compared with NSSDE patients and healthy controls, Dendritic cell density and all morphologic parameters were significantly higher in SSDE group. Dendritic cell density and morphologic parameters, except for dendritic cell size, were significantly higher in NSSDE as compared with the controls. *SSDE* Sjogren’s syndrome dry eye, *NSSDE* non-Sjogren’s syndrome dry eye.

**Figure 2:**
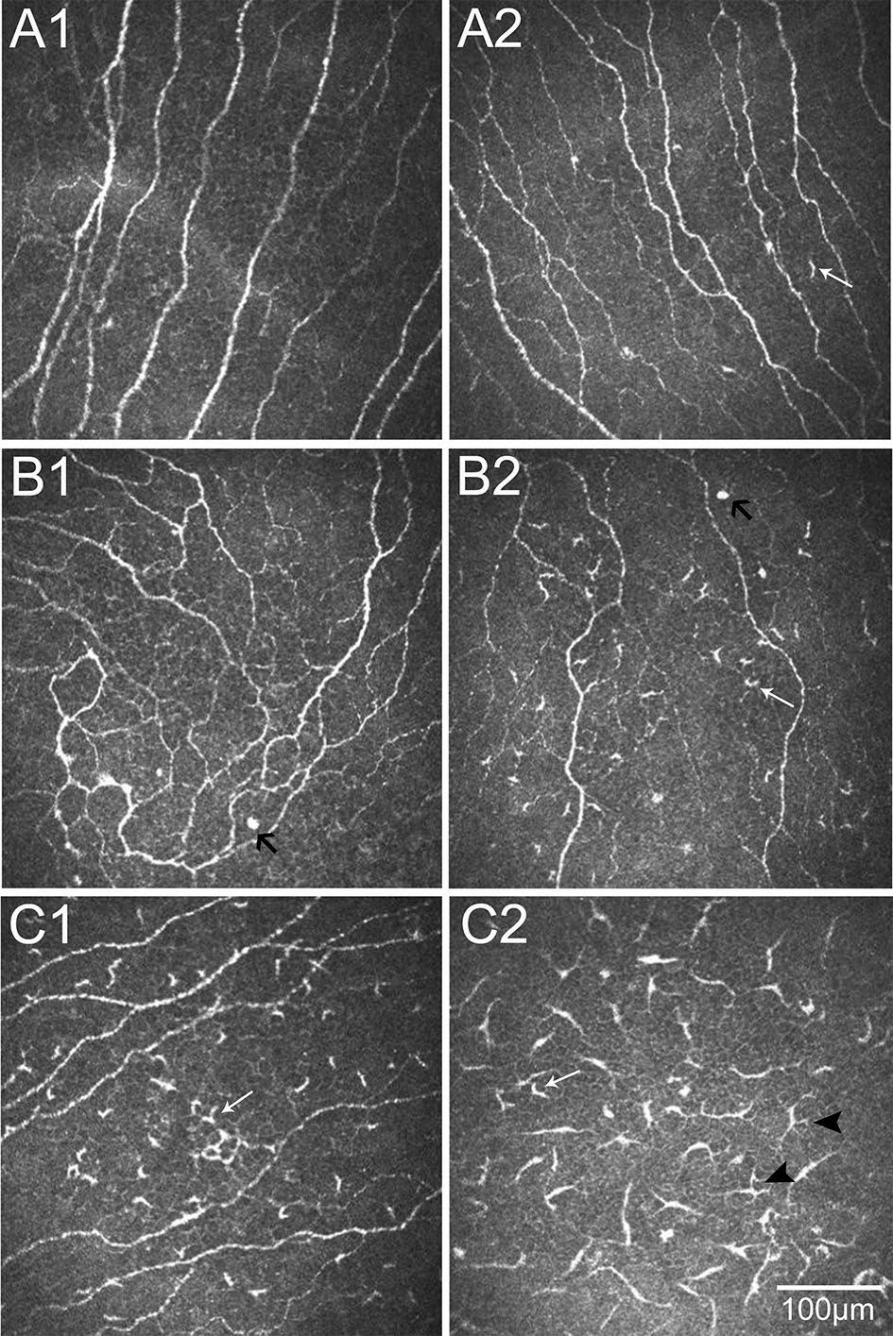
Representative in vivo confocal microscopic images of corneal epithelial dendritic cell (DC) in three groups. (**A**) Central cornea of a healthy volunteer, showing without DC (**A1**) and small DCs with two processes (white arrow) (**A2**). (**B**) Central cornea of a 39-year-old female patient with non-Sjogren’s syndrome dry eye (NSSDE), showing hyperreflective DCs without dendritic process (black arrow in **B1** and **B2**) and a higher density of DCs (**B2**) with two processes (white arrow). (**C1** and **C2**) Central cornea of a 43-year-old female patient with Sjogren’s syndrome dry eye (SSDE), showing a great number of DCs with two (white arrow) and longer processes (arrowhead).

Table 2 and Fig. 1 showed the IVCM parameters for DC in three groups. The DC density (202.34 ± 43.25 cells/mm^2^), DC size [3 (2, 3)], number of dendrites (3.84 ± 0.44), and DC field (393.21 ± 57.13 μm^2^) in the SSDE group (Fig. 1 and 2C1–C2) were significantly higher as compared with the NSSDE (Figs. 1 and 2B1–B2) and healthy control group (Figs. 1 and 2A1–A2) (all *P* < 0.001). Compared with the healthy control group, the patients with NSSDE had a higher DC density (20.25 ± 8.44 vs. 67.74 ± 15.53 cells/mm^2^, respectively, *P* < 0.001), higher number of dendrites per cell (2.15 ± 0.08 vs. 3.23 ± 0.19, respectively, *P* = 0.043), and larger DC field (128.48 ± 19.61 vs. 196.11 ± 69.45 μm^2^, respectively, *P* = 0.023). However, the difference in DC size between the healthy controls and NSSDE group was not statistically significant [2 (1, 2) vs. 2 (1, 3), respectively, *P* = 0.076].

**Table 2:** The in vivo confocal microscopy parameters for dendritic cell among three groups.

Group	DC density, cells/mm^2#^	DC size^$^	DC dendrites, number/cell^#^	DC field, μm^2#^
SSDE (n = 28)	202.34 ± 43.25	3 (2, 3)	3.84 ± 0.44	393.21 ± 57.13
NSSDE (n = 33)	67.74 ± 15.53	2 (1, 3)	3.23 ± 0.19	196.11 ± 69.45
Control (n = 30)	20.25 ± 8.44	2 (1, 2)	2.15 ± 0.08	128.48 ± 19.61
Difference between SSDE and NSSDE	*P* < 0.001	*P* < 0.001	*P* < 0.001	*P* < 0.001
Difference between SSDE and control	*P* < 0.001	*P* < 0.001	*P* < 0.001	*P* < 0.001
Difference between NSSDE and control	*P* < 0.001	*P* = 0.076	*P* = 0.043	*P* = 0.023
Difference among three groups	*P* < 0.001	*P* < 0.001	*P* < 0.001	*P* < 0.001

*DC* dendritic cell, *SSDE* Sjogren’s syndrome dry eye, *NSSDE* non-Sjogren’s syndrome dry eye;

^#^Mean ± standard deviation; ^$^Median (25% quantile, 75% quantile).

### Correlation between Clinical Manifestation and DC Density and Morphology

To determine whether there is a correlation between DC density/morphology and systematic/ocular manifestations of SS, we performed Spearman’s rank order correlation analysis of clinical data and IVCM parameters. The correlation coefficients (r) and *P* values were shown in Table 3. The results showed that there were positive correlations between salivary gland biopsy and DC density (r = 0.499), DC size (r = 0.402), DC dendrites (r = 0.465) as well as DC field (r = 0.473) (all *P* < 0.001). Positive correlations were also noted among anti-SSA52, anti-SSA, anti-SSB and DC dendrites (r = 0.384, r = 0.399, r = 0.329); anti-SSA52, anti-SSA and DC size (r = 0.332, r = 0.357); as well as anti-SSA and DC field (r = 0.302) (all *P* < 0.001). OSDI scores correlated positively with DC density (r = 0.336, *P* = 0.007), DC dendrite (r = 0.311, *P* = 0.001) and DC field (r = 0.306, *P* = 0.001). Meanwhile, ST, TMH, and TBUT showed inverse correlations with the DC density (r = − 0.405, *P* = 0.004; r = − 0.386, *P* = 0.006; r = − 0.576, *P* = 0.018) and DC field (r = − 0.331, *P* = 0.007; r = − 0.286, *P* = 0.035; r = − 0.431, *P* = 0.004). Moreover, TBUT also demonstrated an inverse correlation with DC dendrites (r = − 0.386, *P* = 0.007). The OSS score showed marked positively correlations with DC density (r = 0.524, *P* = 0.032), DC dendrites (r = 0.587, *P* = 0.001), and DC field (r = 0.589, *P* = 0.001), however, a weak positive correlation with DC size (r = 0.234, *P* = 0.006).

**Table 3:** Correlations between DCs density/morphology and clinical characteristics.

	DC density	DC size	DC dendrites	DC field
**Age**				
r	0.195	0.178	0.196	0.120
*P*	0.123	0.068	0.073	0.165
**Sex**				
r	− 0.048	− 0.017	− 0.060	− 0.057
*P*	0.576	0.838	0.483	0.504
**Salivary gland biopsy**				
r	0.499**	0.402**	0.465**	0.473**
*P*	0.000	0.000	0.000	0.000
**Anti-SSA52**				
r	0.271**	0.332**	0.384**	0.291**
*P*	0.001	0.000	0.000	0.001
**Anti-SSA**				
r	0.268**	0.357**	0.399**	0.302**
*P*	0.001	0.000	0.000	0.000
**Anti-SSB**				
r	0.227**	0.296**	0.329**	0.284**
*P*	0.007	0.000	0.000	0.001
**OSDI**				
r	0.336**	0.289**	0.311**	0.306**
*P*	0.007	0.001	0.001	0.001
**ST**				
r	− 0.405**	− 0.177	− 0.152	− 0.331**
*P*	0.004	0.069	0.078	0.007
**TMH**				
r	− 0.386**	− 0.129	− 0.163	− 0.286*
*P*	0.006	0.074	0.075	0.035
**TBUT**				
r	− 0.576*	− 0.164*	− 0.386**	− 0.431**
*P*	0.018	0.035	0.007	0.004
**OSS**				
r	0.524*	0.234**	0.587**	0.589**
*P*	0.032	0.006	0.001	0.001

*DC* dendritic cell, *anti-SSA* anti-Sjogren's syndrome A, *anti-SSB* anti-Sjogren's syndrome B, *OSDI* ocular surface disease index, *ST* Schirmer’s test, *TMH* tear meniscus height, *TBUT* tear film breakup time, *OSS* ocular staining score.

r is shown for all significant correlations in bold.

*P < 0.05; **P < 0.01.

**Figure 3:**
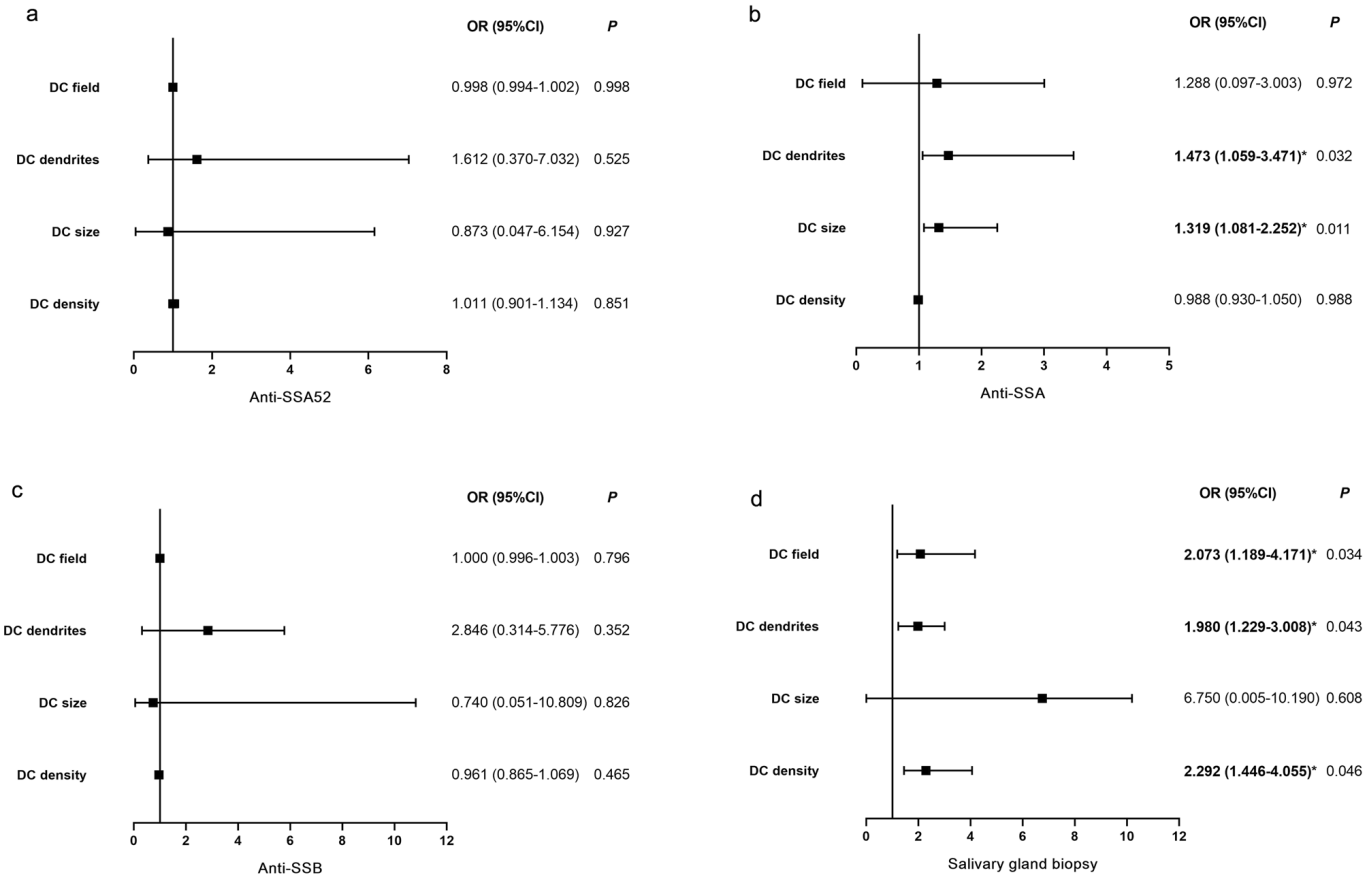
Logistic regression models: associations between DC parameters and anti-SSA52 (**a**), anti-SSA (**b**), anti-SSB (**c**) and salivary gland biopsy (**d**). OR (95% CI) indicates the risk of positive anti-SSA antibody and salivary gland biopsy enhanced when DC density, size, dendrites and field increased. *DC* dendritic cell, *anti-SSA* anti-Sjogren's syndrome A, *anti-SSB* anti-Sjogren's syndrome B, *OR* odds ratio, *CI* confidence interval; *P < 0.05.

The associations between DC density/morphology and systematic manifestations (salivary gland biopsy and serum antibodies) in SSDE are shown in Fig. 3. After multivariable adjustment, DC density showed an odd ratio (OR) 2.292 [95% confidence interval (CI) 1.446–4.055, *P* = 0.046] for positive salivary gland biopsy. DC size had an OR 1.319 (95% CI 1.081–2.252, *P* = 0.011) for positive anti-SSA. DC dendrites had an OR 1.980 (95% CI 1.229–3.008, *P* = 0.043) for positive salivary gland biopsy and OR 1.473 (95% CI 1.059–3.471, *P* = 0.032) for positive anti-SSA. DC field presented an OR 2.073 (95% CI 1.189–4.171, *P* = 0.034) for positive salivary gland biopsy. No significant association of DC parameters with anti-SSA52 and anti-SSB was observed (all *P* > 0.05).

The effects of DC parameters on ocular features in SSDE patients are shown in Table 4. Significant associations were found between higher OSDI scores and increased DC density [β = 0.316 (95% CI 0.186–0.519), *P* = 0.049, per 1 cells/mm^2^ increase], increased DC dendrites [β = 0.144 (95% CI 0.072–0.315), *P* = 0.037, per 1 number/cell increase], and increased DC field [β = 0.053 (95% CI 0.021–0.086), *P* = 0.004, per 1 μm^2^ increase]. Significant associations were shown between increased OSS and higher DC density [β = 0.260 (95% CI 0.146–0.475), *P* = 0.016], DC dendrites [β = 0.259 (95% CI 0.117–0.598), *P* = 0.042], and DC field [β = 0.015 (95% CI 0.010–0.018), *P* = 0.003]. Higher DC density was also associated with decreased ST [β = -0.170 (95% CI − 0.387 ~ − 0.054), *P* = 0.002] and TBUT [β = − 0.125 (95% CI − 0.392 ~ − 0.072), *P* = 0.045]. Higher DC field was associated with decreased ST [β = − 0.012 (95% CI − 0.018 ~ − 0.010), *P* = 0.001] and TMH [β = − 0.008 (95% CI − 0.011 ~ − 0.004), *P* = 0.023].

**Table 4:** Multivariate linear regression models: associations between DC parameters and ocular features in SSDE.

	DC density (per 1 cells/mm^2^)		DC size (per 1 grade)		DC dendrites (per 1 number/cell)		DC field (per 1μm^2^)	
	Estimate (95% CI)	*P*	Estimate (95% CI)	*P*	Estimate (95% CI)	*P*	Estimate (95% CI)	*P*
OSDI	0.316 (0.186–0.519)*	0.049	3.035 (0.206–10.319)	0.300	0.144 (0.072–0.315)*	0.037	0.053 (0.021–0.086)**	0.004
ST	− 0.170 (− 0.387 ~ − 0.054)**	0.002	0.650 (− 0.243–1.544)	0.140	0.015 (− 0.019–0.049)	0.357	− 0.012 (− 0.018 ~ − 0.010)**	0.001
TMH	0.001 (− 0.001–0.003)	0.147	− 0.004 (− 0.054–0.047)	0.873	0.012 (− 0.029–0.052)	0.550	− 0.008 (− 0.011 ~ − 0.004)*	0.023
TBUT	− 0.125 (− 0.392 ~ − 0.072)*	0.045	0.118 (− 1.911–1.947)	0.891	0.007 (− 0.063–0.077)	0.835	0.001 (− 0.002–0.003)	0.751
OSS	0.260 (0.146–0.475)*	0.016	3.078 (− 3.102–9.258)	0.302	0.259 (0.117–0.598)*	0.042	0.015 (0.010–0.018)**	0.003

*DC* dendritic cell, *SSDE* Sjogren’s syndrome dry eye, *OSDI* ocular surface disease index, *ST* Schirmer’s test, *TMH* tear meniscus height, *TBUT* tear film breakup time, *OSS* ocular staining score, *CI* confidence interval.

The Estimate (95% CI) is shown for all significant correlations in bold.

*P < 0.05.

## Discussion

Corneal epithelial APCs, particularly DCs, play an essential role in corneal immune surveillance and homeostasis, including the initiation and induction of ocular surface immune reactivity and tolerance^13,28,29^. Development of IVCM made it feasible to investigate and quantify DCs alterations in various ocular surface diseases, such as DED^7,14,24,30–33^, keratitis^18^, contact lens wear^34,35^, and corneal graft rejection^36^, both in DC density and morphology. This study showed significant increases in DC density and morphologic parameters (size, dendrite number, and field) in aqueous tear-deficient DE patients with or without SS. Furthermore, as compared with NSSDE patients, increased DC density with higher activation state (higher DC size scores) and larger DC fields with more dendrites were noted in patients with SSDE. Additional, DC density and morphologic parameters showed marked correlations with systemic severity (salivary gland biopsy and anti-SSA antibody) and ocular surface discomfort (OSDI) as well as damage (ST, TMH, TBUT and OSS). Those results may contribute to stratify patient and evaluate treatment effect.

In our study, the SSDE and NSSDE patients demonstrated differential degrees of increased DC parameters (Table 2, Figs. 1 and 2). Compared with the healthy controls, eyes with both SSDE and NSSDE had significant increases in DC density. The result is consistent with the notion that hyposecretion and immunoinflammatory response are similar in both SS and NSS^1,4^. However, it should be bear in mind that SS is a chronic inflammatory disease with more severe clinical manifestations than NSS^1,4^. Furthermore, comparison of SSDE and NSSDE revealed that DC density was higher in the SSDE subtype (*P* < 0.001). Therefore, corneal immune and inflammatory responses were seen in both subtypes of aqueous tear-deficient DE and were more severe in SSDE. This was in accordance with previous reports, increased DC density was found in eyes with SS compared with non-SS^31^. Furthermore, it was reported by Lin et al. that the density and size of DC increased in the central cornea of DED with and without SS, whereas higher DC density and larger DC size in peripheral corneas were only found in SS patients^7^. In addition, Wakamatsu and coworkers demonstrated that increased DC number was found both in cornea and conjunctiva of patients with SSDE compared with NSSDE^37^.

Corneal DCs will increase and mature throughout the cornea on inflammatory stimulation, with the increased expression of major histocompatibility complex (MHC) class II and co-stimulatory molecules^13,38^. Notably, the upregulation of MHC-II DC has been verified in patients with SS and implicated in this complex autoimmune disorder^39,40^. Interestingly, human leukocyte antigen (HLA)-DR, encoded specific MHC II DC, is upregulated in SSDE and NSSDE patients^41,42^. However, the levels of HLA-DR are significantly higher in SSDE^39,42^. These researches suggested that HLA-DR upregulation and MHC II DC overexpression played essential roles in the pathophysiology of aqueous tear-deficient DE, especially in SSDE. Clinically, upregulated HLA-DR has been acted as an active marker in SSDE, and reduced HLA-DR expression could be observed in experimental testing of the treatment efficacy^43,44^. Since the visibility of DC density and morphology with IVCM, which reflected the corneal immune and inflammatory changes. It may be possible to evaluate the activity of the immune system and the severity of the inflammatory response using IVCM, and help to stratify patient and tailor treatment.

Morphologic parameters of corneal DC are associated with their functional forms^6,7,45^. It is well known that mature DCs are larger with longer processes, whereas immature DCs are smaller ones and lack dendrites^46^. In our study, we found that small DCs with lacked dendritic extensions often seen in healthy controls (Fig. 2A2). Moreover, in the NSSDE group, larger DCs in the central corneal epithelium with more processes increased (Fig. 2B2), and even more so in the SSDE group (Fig. 2C1–C2). However, compared with the healthy controls, eyes with NSSDE group had no significant increase in DC size. This result also suggested that immune and inflammatory responses were more prominent in SS. Our study provided further evidence that DCs participated in aqueous tear deficient DE pathogenesis, especially in SSDE.

Furthermore, the density and morphology of DCs in the central corneal epithelium showed marked positive correlations with ocular surface damage (OSS score), whereas inverse correlations with hyposecretion conditions (ST and TMH) and tear film instability (TBUT). In other words, there were positive correlations between the density and morphology of the DC and eye severity in SS patients. The presence of DCs as the immune sentinel and biomarker of the corneal immune response has been identified both in animal and human studies^13,14,24,47^. The multivariate linear regression analysis of DC parameters with ocular factors also found that OSDI and OSS were independently associated with DC density, DC dendrites and DC field, while ST and TBUT were independently associated with DC density. A recent study by Yang et al.^48^ found a correlation of ocular surface staining with inflammatory cytokines in DED patients, which in agreement with our study that vital dye staining may result in underlying immune activation or vice versa. Aggarwal et al.^24^ also found TBUT was associated with DC dendrites, however, there was no association between DC parameters and OSDI/ST in DED patients. These discrepancies may be due to the different participants enrolled and SSDE patients showed more serious ocular discomfort and injury than NSSDE. These findings demonstrate that DC parameters may be a useful biomarker to help make an objective assessment of the corneal immune response in DED, especially in SSDE.

In addition, it is worth noting that the associations between salivary gland biopsy and DC density (OR = 2.292), DC dendrites (OR = 1.980) as well as DC field (OR = 2.073), between anti-SSA antibody and DC size (OR = 1.319) as well as DC dendrites (OR = 1.473). These results demonstrated that increased DC density and morphology changes could result in higher risks of positive salivary gland biopsy and anti-SSA antibody in SS. Previous studies have identified that DCs were significantly increased in the central corneal epithelium^13,47^, and simultaneously MHC class II molecules were also upregulated in salivary gland in SS patients^7,49^. With continuous injury to the system and ocular surface in SS patients, the cycle of antigen recognition, immune response, and inflammatory damage are perpetuating and exacerbating^39^. Thus, the density and the morphologic features of DCs may provide an index for evaluating immunoinflammatory status in SS patients. Since the salivary gland biopsy and serum antibody tests are not feasible during repeated follow-ups in SS patients, the observation of DC density and morphologic parameters with IVCM may be used as an approach to assess the activity and severity of immune and inflammation in SS and may also facilitate to early diagnose as well as monitor the therapeutic effect.

Resident corneal immune cells are considered long-lived and can maintain corneal homeostasis^38,47^. With immune and inflammatory stimuli, corneal DCs, which at least in part recruited from the blood, are increased and activated^38,47,50^. The blood-derived DCs initially reach the limbus and later migrate to peripheral and central cornea^20,38,47^. Both in humans and rodents, the DCs have been reported to reside very close to the corneal nerves^47,51^. According to the neuro-immune interaction, corneal nerves play an important role in DCs morphological changes and kinetic alterations^47^. In this present study, there was a clear difference on corneal nerve density and morphology among SSDE, NSSDE and healthy controls (shown in Fig. 2). Our study provided further evidence that there was a possible relationship between nerves and DCs. The impacts of corneal nerves on the initiation and regulation of DCs both in health and disease, and the underlying mechanisms warrant further in-depth researches.

Inevitably, our study has several limitations. First, the evaporative DE was not included in this study, which also showed DC alterations in previous study^6^. Moreover, the meibomian gland may also affect the DC density and morphology and should be demonstrate in further research. Second, only the central cornea was investigated in our study since previous research has found that DC changes were more obvious in the central than the peripheral cornea in SSDE^7^. The DC parameters in the peripheral cornea/conjunctiva cannot be extrapolated. Currently a study with larger patients is underway to assess the DC changes both in central and peripheral cornea. Third, further larger sample investigations are needed to determine the efficacy of using DC density and morphology to evaluate the therapeutic effects of DED.

Nevertheless, this present study provides a quantitative description of DC density and morphology in DE patients with or without SS. Moreover, DC parameters showed significant correlations with activity and severity of SS and DED. Additionally, the demonstration of differential responses in DC parameters may further elucidate the pathogenesis of this complex disease.

In summary, the SSDE are characterized by increased DC density, higher DC activation, more dendrites and larger fields. Such DC alterations could contribute to activity and severity of SS and can be detected and quantified by IVCM objectively, allowing its use in evaluating pathogenesis and guiding clinical treatment.

## Data Availability

The datasets used and/or analyzed during the current study are available from the corresponding author on reasonable request.
